# Intravitreal Inflammation: From Benchside to Bedside

**DOI:** 10.1155/2013/758035

**Published:** 2013-01-15

**Authors:** Mario R. Romano, John Christoforidis, Ahmed M. Abu El-Asrar

**Affiliations:** ^1^Department of Ophthalmology, Istituto Clinico Humanitas, Rozzano, 20089 Milan, Italy; ^2^Department of Ophthalmology, College of Medicine, The University of Arizona, Tucson, AZ 85711, USA; ^3^Department of Ophthalmology, College of Medicine, King Saud University, Riyadh 11451, Saudi Arabia

Inflammation plays a major role in the formation and in the progression of sight-threatening chorioretinal diseases such as diabetic retinopathy (DR), proliferative vitreoretinopathy (PVR), uveitis, and age-related macular degeneration (AMD). A greater understanding of the underlying pathological mechanisms is necessary for the development of better therapeutic agents and relies on the analysis of clinical specimens as well as on animal models. Contrary to the retina, the vitreous humour (VH) is a transparent gel that fills the posterior chamber of the eye and can be sampled without causing visual loss. In recent years, advances in the analysis of VH samples have highlighted new biological mechanisms of long-known diseases and have improved the accuracy of diagnostic procedures.

In this special issue, we report how the VH findings at the benchside can be translated to the bedside, and how this may help clinical practice. The papers have been contributed by a number of experts in the field and include both review articles that provide an overview of the work conducted to date, as well as original articles reporting recent discoveries and innovations. In order to highlight the translational relevance of VH analyses, most of the papers are focused on a specific disease entity. We hope that this series of manuscripts will be beneficial for clinicians in their diagnostic and therapeutic approaches towards intravitreal inflammatory conditions and for researchers in appreciating some of the recent innovations and their clinical implications in this field. Each of the manuscripts in this series is briefly highlighted as follows.

The paper by M. Angi et al. “*Proteomic analyses of the vitreous humour*” describes how to correctly collect and handle VH specimens and presents a clear workflow for proteomic analyses. This is significant since the VH is not a straightforward tissue to analyze due to its viscous consistency. Proteomic technologies have dramatically evolved over the past years, allowing identification of an increasing number of disease-specific proteins in the VH. Moreover, recent proteomic studies on the VH from animal models of autoimmune uveitis have highlighted new pathways associated to autoimmune triggers and intravitreal inflammation that could become the targets for much needed therapies.

Another example of the usefulness of proteomic analyses of the VH in translational research is presented by O. Simó-Servat et al. in “*Usefulness of the vitreous fluid analysis in the translational research of diabetic retinopathy*” who applied fluorescence-based difference gel electrophoresis (DIGE), as well as flow cytometry, to identify new candidates involved in the inflammatory process that occurs in DR. The authors provide evidence supporting the role of proinflammatory mediators such as cytokines (i.e., IL-1*β*, IL-6, IL-8, and TNF*α*), chemokines (i.e., MCP-1, SDF-1, and IP-10), and adhesion molecules (i.e., VCAM, ICAM-1, and VAP-1) in the pathogenesis of DR. Such persistent low-grade inflammation contributes to the damage of the internal blood-retinal barrier and to the development of proliferative diabetic retinopathy (PDR).

A. M. Abu El-Asrar et al. in “*Osteopontin and other regulators of angiogenesis and fibrogenesis in the vitreous from patients with proliferative vitreoretinal disorders*” and “*High-mobility group box-1 and endothelial cell angiogenic markers in the vitreous from patients with proliferative diabetic retinopathy*” investigate the role of osteopontin and other regulators of angiogenesis and fibrogenesis, such as high-mobility group box-1 (HMGB1) and connective tissue growth factor (CTGF) in the pathogenesis of proliferative vitreoretinal disorders with a concomitant increase of antifibrogenic pigment epithelium-derived factor (PEDF) levels in the VH. Moreover, the authors report that HMGB1, soluble vascular endothelial-cadherin (sVE-cadherin), and soluble endoglin (sEng) regulate the angiogenesis of endothelial cells in PDR.

R. dell'Omo et al. in “*Vitreous mediators in retinal hypoxic disease*” describe that serum adiponectin (APN) levels correlate with blood inflammatory marker levels and with DR as response to endothelium dysfunction, indicating the role of APN as endogenous modulator of microvascular function and inflammation.

S. N. Moysidis et al. in “*Mechanisms of inflammation in proliferative vitreoretinopathy: from bench to bedside*” describe the indirect activation of PDGFR*α* by non-PDGFs as trigger that leads to development of PVR. In this pathway, the intracellular reactive oxygen species (ROS) plays a key role, leading to activation of Src family kinases (SFKs) that promote phosphorylation and activation of PDGFR*α*. The ROS could be one of the therapeutic targets of multimodal approach.

D. Gologorsky et al. in “*Therapeutic interventions against inflammatory and angiogenic mediators in proliferative diabetic retinopathy*” report the latest focus of targeted therapies for proliferative diseases through the block of vascular adhesion molecules such as ICAM-1, VCAM-1, inflammatory factors including the interleukins, tumor necrosis factor (TNF), insulin-like growth factor (IGF), and angiopoietins (Ang-2).

Analysis of the VH is a valuable adjunct also for the management of patients with uveitis and especially in the diagnosis of neoplastic diseases masquerading as chronic intraocular inflammation, as reported by E. M. Damato et al. in “*Vitreous analysis in the management of uveitis.*” For example, increased levels of T-cell cytokine, IL-6, in VH is characteristic of uveitis, whereas increased levels of IL-10 and in particular IL-10/IL-6 ratio greater than 1 should prompt cytological analysis for the diagnosis of vitreoretinal lymphoma. The involvement of VH in neoplastic diseases and the pros and cons of performing VH biopsies in the clinical practice are further discussed in the review article by M. Asencio-Duran et al. entitled “*Vitreous diagnosis in neoplastic diseases.*”

J. L. Vallejo-Garcia et al. in “*Role of inflammation in endophthalmitis*” discuss the role of inflammation in infective endophthalmitis, reporting that the damage to the retina in this rare but severe diseases is mediated by the host immune reaction through toll-like receptors, cytokines, HMGB1, and aB-crystallin. A better understanding of the host immune reaction and the cellular pathways leading to tissue damage is also essential to improve clinical outcomes. Corticosteroids are frequently administered with antibiotics but often do not fully control the host immune reaction with consequent visual loss. A novel TLR2 ligand, Pam3Cys, has demonstrated encouraging results when administrated before the onset of endophthalmitis and also when injected in combination with intravitreal antibiotics.

J. B. Christoforidis et al. in “*Intravitreal devices for the treatment of vitreous inflammation*” describe the importance of the modulation of pharmacokinetics in the treatment of chronic intraocular inflammation. Long-term treatments are currently provided by drug-delivery devices, which include nonbiodegradable and biodegradable devices. The therapeutic agents that can be delivered are ganciclovir, fluocinolone acetonide, triamcinolone acetonide, and dexamethasone. The next small-scale biodegradable devices already described are liposomes, microspheres, and nanoparticles from 0.01 to 1,000 *μ*m in diameter.

J. B. Christoforidis et al. in “*Systemic treatment of vitreous inflammation*” also report that many classes of systemic drugs may be used alone or in combination to control intraocular inflammation while closely monitoring side effects. Many of these inflammatory disorders require long-term treatment, and hence steroid-sparing agents, including antimetabolites, alkylating agents, and biological agents, are being used.

The emerging topic of sterile endophthalmitis is presented by J. Marticorena et al. in “*Sterile endophthalmitis after intravitreal injections.*” It is an infrequent complication of intravitreal injections and seems to develop in the context of the off-label use of drugs that have not been conceived for intravitreous administration. Sterile inflammation secondary to IVTA and IVB share many characteristics, such as the acute and painless vision loss present in the vast majority of the cases.

Inflammation also plays a major role also in the aging retina, where free radicals and oxidized lipoproteins are considered to be major causes of tissue stress. F. Parmeggiani et al. in “*Mechanism of inflammation in age-related macular degeneration*” report that the consequence is a parainflammation, a chronic status which contributes to initiation and progression of neurodegenerative diseases such as age-related macular degeneration (AMD). The parainflammatory deregulation that is already present in the early stage of AMD may notionally support the preventive employment of agents directed against the immune-inflammatory response in combination with high-dose nutritional supplements.

We sincerely hope that the present special issue may provide useful information to understand the mechanisms, the clinical effects, and the novel treatments of inflammation in which the vitreous is involved.

*Mario R. Romano* *Mario R. Romano* *John Christoforidis* *John Christoforidis* *Ahmed M. Abu El-Asrar* *Ahmed M. Abu El-Asrar*

